# Improving the Management of Outpatients with Heart Failure the IC-MMERSIVE Project

**DOI:** 10.3390/jcm15072530

**Published:** 2026-03-26

**Authors:** Vivencio Barrios, Carlos Escobar, Gonzalo Luis Alonso, Ramón Bover, Maria José Castillo, Román Freixa-Pamias, Raquel López-Vilella

**Affiliations:** 1 Cardiology Department, University Hospital Ramon y Cajal, 28034 Madrid, Spain; 2 Cardiology Department, University Hospital La Paz, 28046 Madrid, Spain; 3 Cardiology Department, University Hospital de Navarra, 31008 Pamplona, Spain; gonzalol.alonso@gmail.com; 4 Cardiology Department, University Hospital Clinico, 28040 Madrid, Spain; ramonbover@yahoo.es; 5 Primary Care Center, La Algaida-Barrio Bajo Sanlúcar de Barrameda, 11549 Cádiz, Spain; mariajosecastillo4@hotmail.com; 6 Cardiology Department, Hospital Sant Joan Despí Moisès Broggi, 08970 Sant Joan Despí, Barcelona, Spain; rfreixap@gmail.com; 7 Cardiology Department, University Hospital La Fe, 46026 Valencia, Spain; cune10@hotmail.com

**Keywords:** cardiology, diagnosis, heart failure, treatment

## Abstract

**Objectives**: Design strategies to improve management, outcomes, and quality of life for people with heart failure (HF) in Spain through the identification of areas of improvement regarding diagnosis, treatment, comorbidities, progression of disease and healthcare coordination between specialists. **Methods**: IC-MMERSIVE project was developed by the Cardiology and Primary Care Integration Working Group of the Spanish Society of Cardiology. The project included a pre-session survey for participants, face-to-face sessions led by a clinical cardiologist, and post-session questionnaires for the moderator and for participants. A web platform was created to host program content and resources and electronic forms for data collection and analysis. **Results**: A total of 1186 physicians (80.5% cardiologists) participated in 144 face-to-face sessions throughout Spain. When patients are at risk for HF (HF stage B), 78.9% of respondents said they proactively search for HF. Only 38.0% were familiar with and applied the IC-BERG study questions designed to detect falsely stable patients. Specific protocols for optimizing and implementing the four pharmacologic pillars of treatment for HF were used by 51.6% of participants, 53.9% had protocols to reach the guideline-recommended target doses, and 25.6% reported no nursing involvement. Structured follow-up was conducted in 53.9% of cases. Even though 63.0% used shared single medical records, the connection between specialized HF consultations and healthcare centers was occasional in 72.1% of cases. **Conclusions**: There is marked room to improve HF management in daily clinical practice. These findings highlight specific actionable gaps in HF management and support the need for structured, multidisciplinary strategies to improve patient outcomes.

## 1. Introduction

Heart failure (HF) represents a major global healthcare issue, affecting an estimated 1% to 2% of the adult population worldwide, with prevalence rising to 4% when undiagnosed cases among older adults are considered. Prevalence increases markedly with age, exceeding 10% in individuals over 70 years. Consequently, HF impacts millions globally, with particularly high rates observed in developed regions such as North America and Europe. This growing prevalence is primarily attributable to demographic shifts toward an aging population, enhanced survival following other cardiovascular conditions, and the persistent influence of risk factors including hypertension and diabetes [1,2,3].

HF remains notably underdiagnosed in clinical practice, particularly in early stages and mainly in primary care, with underdiagnosis rates sometimes surpassing 60%. This challenge arises largely due to symptom overlap with other conditions such as pulmonary diseases or obesity, as well as the diagnostic complexity of HF with preserved ejection fraction. These diagnostic difficulties can result in delayed or inappropriate treatment, poorer patient outcomes, and increased hospital admissions. The issue is especially pronounced among older adults, women, and individuals with diabetes or overweight, representing missed opportunities for timely diagnosis and intervention [4,5,6].

In clinical practice, HF management encompasses a multifaceted approach involving Guideline-Directed Medical Therapy (GDMT), lifestyle interventions, and device therapies. Despite substantial evidence supporting the four pharmacologic pillars of therapy for HF with reduced ejection fraction (HFrEF), including angiotensin receptor/neprilysin inhibitor (ARNI)/angiotensin-converting enzyme inhibitors (ACEi), beta-blockers, mineralocorticoid receptor antagonists (MRA), and sodium-glucose cotransporter 2 inhibitors (SGLT2i), as well as SGLT2i for HF with preserved ejection fraction (HFpEF), significant undertreatment persists, hindering optimal patient outcomes [7,8]. Key barriers include the intricate nature of comorbidity management, issues with patient adherence, and challenges integrating timely initiation of life-prolonging pharmacotherapies into routine clinical workflows. These factors contribute to delayed titration and underuse of recommended therapies, even though guidelines consistently demonstrate marked improvements in survival and reductions in hospitalizations [9,10,11].

To effectively address this situation, it is essential to gather input from physicians involved in the management of HF patients. Their insights can help to accurately identify gaps in diagnostic and therapeutic practices, enabling targeted improvements to be implemented in clinical care. The IC-MMERSIVE (**I**nsuficiencia **C**ardiaca-**M**ejora del **M**an**E**jo y los **R**e**S**ultados clínicos y de cal**I**dad de **V**ida **E**n la consulta–Heart Failure-Improvement in the Management and Clinical Outcomes and Quality of Life Management In the Consultation-) project was aimed to improve management, outcomes, and quality of life for people with HF in Spain through the identification of areas of improvement regarding diagnosis, therapeutic management, comorbidities, progression of disease and care coordination.

## 2. Methods

The IC-MMERSIVE project was developed by the Cardiology and Primary Care Integration Working Group of the Spanish Society of Cardiology. Within this working group, a dedicated scientific committee was established for the project, comprising six experts: cardiologists and Family Medicine specialists. The scientific committee was responsible for developing content, tools such as checklists, and additional resources to support analysis, reflection, debate, and consensus-building during local sessions conducted throughout Spain and aimed to enhance the management of HF within their respective healthcare areas. The scientific committee consisted of experts in both HF patient management and the methodology of such research.

The project included a pre-session survey for participants (Supplementary Table S1), face-to-face sessions led by a clinical cardiologist, a post-session questionnaire for the moderator (Supplementary Table S2), and another for participants (Supplementary Table S3). A web platform was created to host program content and resources, as well as electronic forms for data collection and analysis.

A pre-session survey was administered to participants to gather information on their initial circumstances and clinical context. The pre-session survey was designed to thoroughly examine all facets of HF, including diagnosis, management, follow-up, and specific subgroups. Following completion of the pre-session surveys by participants, an in-depth analysis was conducted. The report of results, presented as response percentages, was reviewed by the session moderator to assess and analyze the starting point of participants (including barriers and difficulties). Moderators received this data alongside a structured script to guide their sessions, as well as a document for recording key discussion points from each meeting. This data allowed the collection of diverse perspectives, facilitated focused reflection, and supported consensus-building around solutions for improvement.

In selecting local meeting participants, careful attention was paid to ensure representation from each region and Spain overall. Random selection processes were used to enhance representativeness and provide a broader perspective on HF management within the country. Face-to-face sessions were conducted under the guidance of a clinical cardiologist serving as moderator and comprised small groups of approximately 5–6 participants working in the same healthcare area. The primary objective was to facilitate dialogue and discussion among all attendees in order to identify challenges, common areas for improvement, and search for potential solutions related to the overall clinical management of patients with HF. These sessions took place within various health areas and departments across Spain. While clinical cardiologists constituted the principal participants, there other specialists, such as Primary Care Physicians and Internal Medicine practitioners, were also involved according to the specific needs of each area. The moderator led the discussions by focusing on locally relevant issues, informed by analysis of previous survey results.

The post-session questionnaire, intended for completion by the moderator, utilized the same format as the published OPTIMISE-IC project [12]. The questionnaire comprised five sections: care coordination, diagnosis and identification of patients with HF, therapeutic management of HF, comorbidities associated with HF, and disease progression in HF. Finally, the participants completed a post-session questionnaire covering the protocol for optimizing HF treatment, frailty, HF management, follow-up procedures, and the communication route.

The scientific committee subsequently performed a comprehensive evaluation of questionnaire results and session summaries. A descriptive and qualitative analysis of the questionnaires was performed. The results of the questionnaires were shown as absolute values and percentages.

## 3. Results

Between 1 June 2024 and 30 July 2025, a total of 144 face-to-face sessions were conducted throughout Spain (Supplementary Table S4). The total number of physicians who participated in these sessions was 1186. Of those, 308 physicians completed and returned the pre-session survey.

Of the 308 physicians, 248 (80.5%) were cardiologists, mainly general cardiologists, 42 (13.6%) internal medicine specialists, and 18 (5.8%) belonged to other specialties such as general practitioners, nephrologists, or HF nurses. Of the physicians, 156 (50.6%) worked in a third-level hospital, 76 (24.7%) in the reference hospital, 70 (22.7%) in a regional hospital, and 6 (1.9%) in outpatient care. Of the centers, 303 (98.4%) were public and 5 (1.6%) were private.

**Figure 1:**
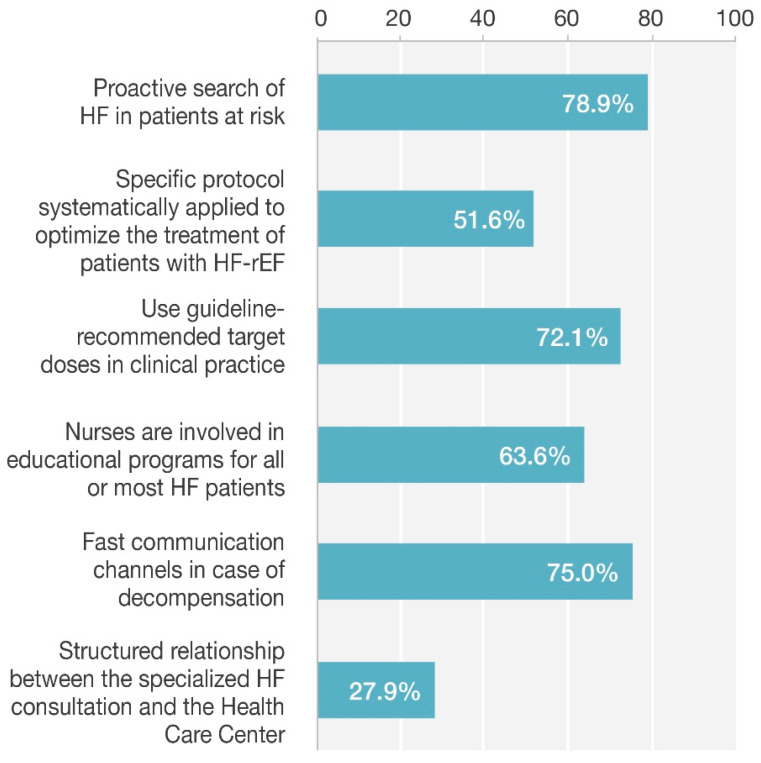
Main results of pre-session survey.

Table 1 and Figure 1 present the results of the pre-session survey. When patients are at risk for HF, 78.9% of respondents said they proactively search for HF, and 79.9% reported being able to perform echocardioscopy during consultations. However, only 38.0% were familiar with and applied the five IC-BERG study questions designed to detect falsely stable patients [13]. Specific protocols for optimizing and implementing the four pharmacological pillars of treatment for HFrEF patients were used by 51.6% of attendees, and 53.9% had protocols to reach the guideline-recommended target doses, although 72.1% prescribed guideline-recommended target doses.

Nursing was involved and provided care for all or most HF patients in 63.6% of cases, while 25.6% reported no nursing involvement. Frailty was assessed in elderly patients by 58.8% of participants, though not with specific scales, and when it was suspected, 52.6% referred patients to specialized units. Iron deficiency was systematically investigated by 88.3% of providers, and management was tailored to individual patient characteristics by 65.3%.

Structured and protocolized follow-up was conducted in 53.9% of cases, while rapid communication channels for decompensation were available in 75.0% of practices. Although 92.5% of participants were aware of criteria for advanced HF, only 34.1% had protocols for identifying and referring these patients to referral units. Even though 63.0% used shared single medical records, the connection between specialized HF consultations and healthcare centers was only occasional in 72.1% of cases.

**Figure 2:**
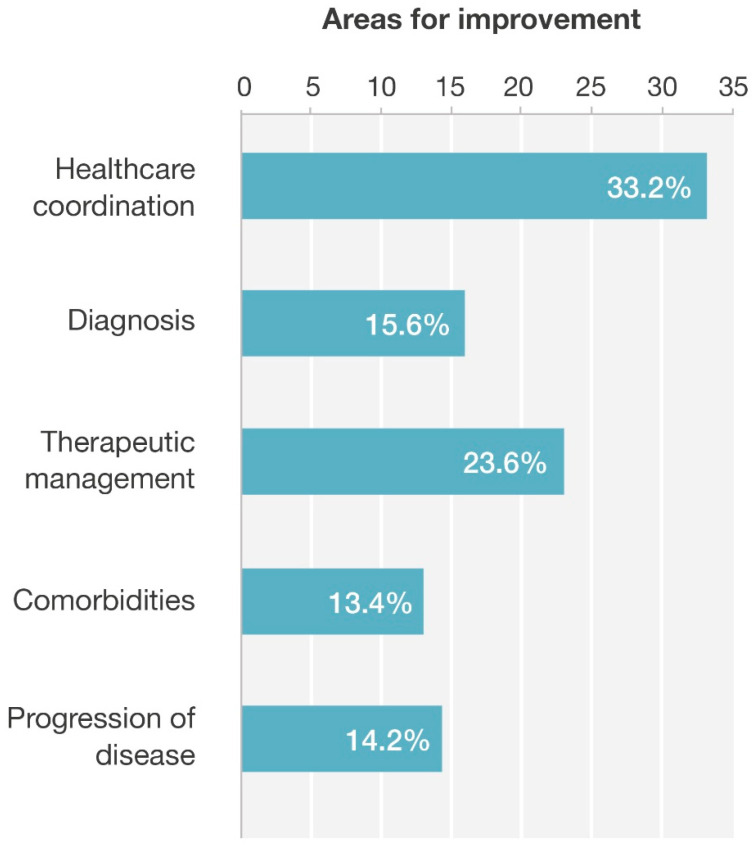
Improvement actions.

A total of 144 moderators participated in the sessions: 134 (93.1%) were cardiologists and 10 (6.9%) were internal medicine specialists. Of the moderators, 68 (47.2%) worked in a third-level hospital, 47 (32.6%) in a regional hospital and 29 (20.1%) in the reference hospital. Of the centers, 141 (97.9%) were public and 3 (2.1%) were private. The moderator, in consensus with the participants, could point out one or more areas for improvement, and one or more lines within the selected area of action. In addition, it was mandatory to indicate at least one area of action and one line within that area of action, and the answers were closed-field. Regarding the improvement actions, 33.2% were focused on healthcare coordination, 23.6% on therapeutic management, 15.6% on diagnosis, 14.2% on disease progression and 13.4% on comorbidities (Figure 2).

Table 2 outlines the actions by category. For healthcare coordination, 22.5% prioritized improving links across different assistive healthcare levels, 20.9% establishing referral and communication protocols, and 16.6% enhancing communication channels and recognizing HF referents in Primary Care. In diagnosis, 28.5% emphasized better proactive identification of at-risk patients (HF stages A or B) and increased universal use of natriuretic peptides. Optimizing the therapeutic plan was important for 25.2%, with 17.4% highlighting improved communication and expert nurse involvement. Proper comorbidity management was marked by the need for individualized follow-up (26.2%), frailty assessment (21.4%), and iron deficiency evaluation (19.8%). For disease progression, participants supported faster communication for decompensation (19.9%), palliative care improvement (16.6%), and greater patient/family education on early signs of decompensation (14.6%).

**Figure 3:**
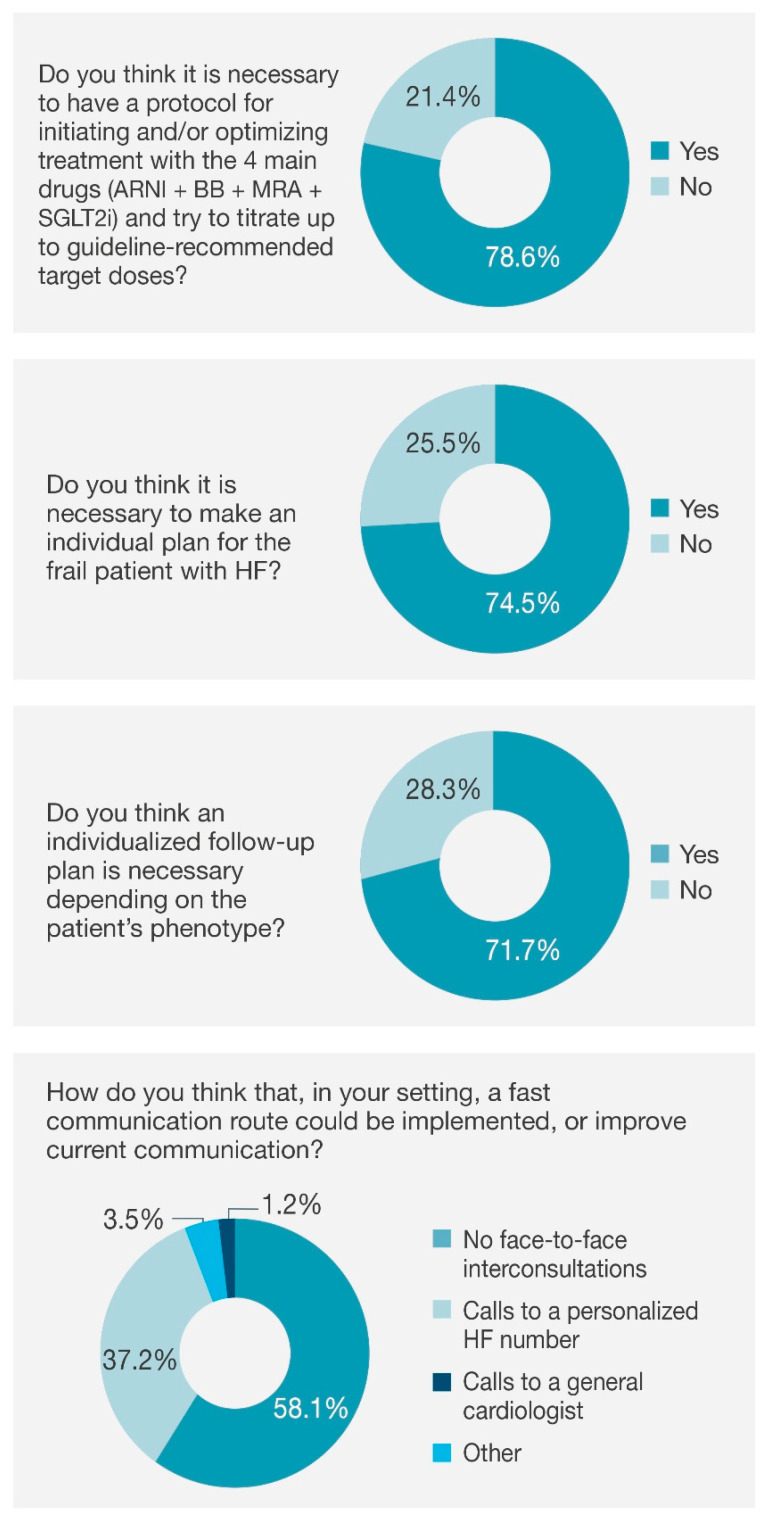
Post-session questionnaire to be completed by participants. ARNI: angiotensin receptor–neprilysin inhibitor; BB: beta blockers; HF: heart failure; MRA: mineralocorticoid receptor antago-nists; SGLT2i: Sodium-Glucose Cotransporter-2 inhibitors.

Among 63 participants who completed the post-session questionnaire, 78.6% supported having a protocol to initiate or optimize HFrEF treatment with the 4 pillars and titrate to target doses, 74.5% favored making individual plans for frail HF patients, 71.7% endorsed tailoring follow-up to patient phenotype, and 58.1% saw value in improving communication through non-face-to-face consultations (Figure 3).

## 4. Discussion

Our research revealed several shortcomings in HF management, such as insufficient detection of at-risk patients, a lack of specific protocols for treatment optimization, limited involvement from nurses, and unstructured follow-up procedures. As a result, we proposed improvements across various areas, including care coordination, therapy management, diagnostics, monitoring disease progression, and addressing comorbidities.

To draw these conclusions, 144 face-to-face sessions were held across Spain, involving 1186 physicians. Out of more than 308 physicians who completed the pre-session survey, about 80% were cardiologists, 14% were internal medicine specialists, and 6% worked in other fields. Additionally, 51% worked in tertiary hospitals. This means that the perspectives of numerous physicians from various specialties and clinical environments were gathered, providing a broad and thorough overview of areas needing improvement in the management of patients with HF.

In our study, approximately 21% of physicians indicated that they did not actively screen for HF, and 20% were unable to perform echocardiography during patient consultations. Many HF patients are not routinely screened or monitored in clinical practice, which increases their risk of death. In the community, numerous cases go undiagnosed due to misdiagnosis, lack of reported symptoms, and gaps in healthcare, making proactive management challenging. For example, a study of 3500 hypertensive women aged 65 and older in Spain found that 26% had undetected clinical HF [14]. Another study revealed that 51% of HF patients were only diagnosed when hospitalized, indicating that many cases are discovered at advanced stages [15]. Additionally, about 40% of HF patients do not see a cardiologist even once a year, a factor associated with higher mortality rates [16]. On the positive side, echocardioscopic screening and improved care pathways can support early diagnosis for at-risk individuals [17]. To address these issues, it is essential to raise awareness among high-risk patients and encourage early diagnosis by increasing clinical suspicion and promoting greater use of echocardiography during consultations.

In Spain, the IC-BERG project developed a brief 5-question survey (i.e., Have you stopped any activities in the past year? Do you experience symptoms of congestion? Do you need to take at least one tablet of furosemide daily? Have you been hospitalized or visited the emergency room for HF decompensation in the past year? Has the NT-proBNP level changed?) to help identify HF patients at risk of decompensation, where optimizing HF treatment is considered essential [13,18,19]. However, our study showed that only 38% of participants were familiar with and applied the five IC-BERG study questions. Additionally, increasing the utilization of natriuretic peptides in primary care may facilitate the early identification of patients at risk for HF [20,21].

Current guidelines delineate the therapeutic strategy for individuals with HFrEF, recommending the prompt initiation of the four foundational drugs: ARNI/ACEi, beta blockers, MRA, and SGLT2i [7,8]. Our findings indicated that only 52% of respondents reported utilizing specific protocols to optimize and implement the four pharmacological pillars of treatment for HFrEF patients, and 54% had established protocols to achieve target dosing of these medications. In a recent retrospective study, only 16.5%, 10%, 31%, and 43% of patients were receiving the target doses of beta-blockers, ACEi/angiotensin receptor blockers/ARNI, MRA, and SGLT2i, respectively. Quadruple therapy was administered to just 21% of patients, and fewer than 1% achieved target dosing for all four medication classes [22]. Establishing protocols that promote the early initiation and optimization of all four pillars of heart failure therapy, including attaining target doses, is essential [23]. In fact, it has been reported that rapid initiation of quadruple therapy is feasible in real-world clinical practice, but a proactive approach is warranted [24].

Nursing care for HF focuses on managing fluid overload, relieving symptoms like fatigue, and encouraging lifestyle changes such as reduced sodium intake, daily weights, and exercise. Key tasks include medication management, patient education, and care coordination to support self-management and prevent readmissions. Despite these advantages, it is noteworthy that one in four respondents indicated an absence of nursing involvement. In this regard, emphasizing enhanced communication and specialized nurse participation appears essential for optimizing the management of patients with HF [25,26]. Furthermore, involvement of additional healthcare professionals may facilitate improved management strategies for these patients. Notably, published evidence indicates that pharmacists can contribute significantly to the early identification of such individuals, thereby reducing time to diagnosis, and play a key role in optimizing HF management [27].

Structured and protocolized follow-up in HF has been shown to markedly reduce hospital readmissions, improve patients’ quality of life, and enhance adherence by shifting from episodic to proactive disease management [28,29]. Unfortunately, this was performed only in 54% of cases. Sharing individual medical records and strengthening links between specialized HF consultations and healthcare centers would enhance care coordination and support protocol implementation in clinical practice.

Proper management of comorbidities is essential for patients with HF. Frailty affects more than half of HF patients and is linked to a 1.5 to 2 times higher risk of death and hospitalization compared to non-frail individuals. It is especially common in those with worsening HF, impacting over 50% of hospitalized HF patients [30]. Our analysis revealed that frailty was evaluated in 59% of elderly patients, highlighting the need for greater efforts to accurately identify and manage frailty in those with HF.

On the other hand, iron deficiency is common in HF, affecting up to half of patients. It worsens fatigue, breathlessness, reduces exercise capacity, and leads to more hospitalizations [31]. In our study, although iron deficiency was systematically investigated by 88% of providers, only management strategies were individualized based on patient characteristics in 65% of cases. This suggests that while awareness of this comorbidity among HF patients appears to be adequate, effective management still falls short of optimal standards.

This study has certain limitations. First, despite efforts being performed to increase the representativeness of the participants, it reflects the opinions and experiences of the physicians who took part in the survey. However, the large number of participants and their wide distribution across Spain may provide a representative view of how HF patients are managed throughout the country. While the pre-session survey had robust participation (*n* = 308), the response rate for the post-session questionnaire was lower (*n* = 63). This subset of respondents might represent highly motivated physicians, potentially introducing a selection bias. Therefore, conclusions drawn specifically from the post-session data regarding protocol implementation preferences should be interpreted with caution, although they reflect the views of engaged clinicians. Unlike other surveys that only include experts, this study allowed participation from all types of physicians, offering a more realistic perspective on medical practice in Spain. Nevertheless, the conclusions drawn from this study apply only to settings similar to those found in Spain. Finally, there may be important discrepancies in the management of HF in the cardiology specialist and general physicians, and a subgroup analysis would be of interest. Unfortunately, the number of physicians belonging to other specialties such as general practitioners was too low to be able to draw relevant conclusions.

## 5. Conclusions

In summary, although there have been significant advancements in the diagnosis and treatment of HF, considerable gaps remain in the management of this population, contributing to an elevated risk of complications. These findings highlight specific actionable gaps in HF management and support the need for structured, multidisciplinary strategies to improve patient outcomes. The project offers practical insights that may help standardize HF care across regions and accelerate implementation of guideline-directed therapies. Enhancing healthcare coordination, optimizing therapy management, advancing diagnostic capabilities, improving disease monitoring, and systematically addressing comorbidities are essential steps toward mitigating these challenges.

## Figures and Tables

**Table 1:** Pre-session survey.

Question	Response	308 (100%)
1. When a patient at risk of developing HF is attended, is a proactive search for HF or pre-HF (ACC-AHA Stages A and B) performed?	NO YES	65 (21.1%) 243 (78.9%)
2. In relation to the evaluation of LVEF, do you feel qualified and equipped to perform an echocardioscopy and thus focus the treatment?	YES NO	246 (79.9%) 62 (20.1%)
2.1. If you DO NOT take it, indicate the waiting time to receive this evaluation	○ <15 days. ○ <1 month. ○ 1–3 months. ○ more than 3 months.	8 (12.9%) 22 (35.5%) 25 (40.3%) 7 (11.3%)
3. Do you know the 5 questions of the IC-BERG study for the detection/identification of the falsely stable patient?	I do NOT know them. I do know them and apply them in the consultation. YES, I know them, but I do not apply them/I cannot apply them in consultation.	167 (54.2%) 117 (38.0%) 24 (7.8%)
3.1. If you know them, which of them helps you the most in your real clinical practice (multiple choice)	Have you stopped doing any activity such as going outside, carrying groceries, climbing the same flights of stairs without stopping, or making things at home, in the last year? Do you have symptoms of congestion? (Weight gain, jugular ingurgitation, ankle edema, sleeping with more pillows) Do you need to take ≥ 1 tablet of furosemide a day? Have you been admitted to or visited the emergency room for HF decompensation in the last year? Has the NT-proBNP levels changed?	68 (27.8%) 68 (27.8%) 35 (14.3%) 48 (19.6%) 26 (10.6%)
4. In your consultation, service or unit, do you have a specific protocol with a therapeutic plan to optimize the patient with HFrEF with the 4 main drugs (ARNI + BB + MRA + SGLT2i)?	YES, and it is systematically applied. YES but I DO NOT apply it systematically. We do NOT have a specific protocol.	159 (51.6%) 30 (9.7%) 119 (38.6%)
5. If a protocol is available, does the protocol take into account the use of guideline-recommended target doses?	YES NO Not applicable: we do not have a protocol.	166 (53.9%) 19 (6.2%) 123 (39.9%)
6. Do you use guideline-recommended target doses in clinical practice? If they are not used, indicate the reasons (multiple choice)	Yes, I use guideline-recommended target doses. I do not prescribe them because I do not think they are essential. I do not prescribe them because our protocol does not contemplate them. I do not use them because the patient cannot tolerate them. I do not use them: they are referred to another unit, and I do not perform the drug titration.	235 (72.1%) 2 (0.6%) 1 (0.3%) 61 (18.7%) 27 (8.3%)
7. In your consultation, is nursing trained in educational programs for patients and caregivers involved in HF?	NO, nursing is not involved in educational programs in my consultation. Nursing is involved and nursing care (assessment, health education and advice) is applied to all or most HF patients. Nursing is involved but nursing care (assessment, health education and advice) is NOT applied to all or most HF patients.	79 (25.6%) 196 (63.6%) 33 (10.7%)
7.1. What barriers do you think exist for its application? (multiple choice)	Education. Consultation time. The assigned nursing tasks do not have this. The patient is referred to a different unit/service where it is performed. Others (open field).	12 (20.7%) 24 (41.4%) 15 (25.9%) 4 (6.9%) 3 (5.2%)
8. Is frailty assessed in patients over 75 years of age in your consultation?	YES, it is assessed WITH previously validated scales. YES, it is assessed but WITHOUT scales: it is a subjective assessment by the cardiologist and/or nurse. NOT usually evaluated.	83 (26.9%) 181 (58.8%) 44 (14.3%)
9. If the patient is considered to be fragile, how is follow-up performed?	The patient is referred to a specialized unit (Internal Medicine or Geriatrics). Follow-up continues in Cardiology. Follow-up is referred to Primary Care.	162 (52.6%) 117 (38.0%) 29 (9.4%)
10. Returning to the protocol or the assessment of the patient with HF. Is iron deficiency taken into account? Is it done in a structured way?	NO YES	36 (11.7%) 272 (88.3%)
10.1. If yes, how often?	○ It is not structured and depends on the patient and the appointment capacity of the consultation. ○ 6 months. ○ 1 year. ○ More than 1 year. ○ Only if symptoms.	136 (50.0%) 106 (39.0%) 28 (10.3%) 2 (0.7%) 0 (0%)
11. What do you do with patients once iron deficiency has been diagnosed and treated?	Routine follow-up of patients with HF. Routine follow-up and fecal occult blood screening. Referral or consultation for the study of iron deficiency in Internal medicine or Digestive specialist. Individualization according to the patient and his/her characteristics.	65 (21.1%) 20 (6.5%) 22 (7.1%) 201 (65.3%)
12. Does the treatment protocol take into account the need to determine renal function and ions when initiating and/or modifying doses of RAASi? Are patients with CKD considered in a special way?	Yes, it is included in the protocol and considers patients with CKD in a special way. Yes, it is included in the protocol, although patients with CKD are not considered in a special way. I do not have a protocol, but I do and I take into account patients with CKD in a special way. I do not have a protocol and I am not specifically prepared to do it.	155 (50.3%) 23 (7.5%) 128 (41.6%) 2 (0.6%)
13. Is there a follow-up protocol in your consultation/unit/service that includes nursing care and the frequency and content of follow-up visits according to comorbidities and/or frailty and the relationship with (apply as the case may be) Cardiology, Primary Care, Internal Medicine, Geriatrics or other specialties?	YES NO	166 (53.9%) 142 (46.1%)
14. Does the patient treated in your consultation have fast communication channels in case of decompensation?	YES NO	231 (75.0%) 77 (25.0%)
14.1. If fast routes are available, who does the patient contact? Is contact direct or indirect through another agent (e.g., Primary Care or Emergency department)?	○ Direct contact with my consultation (doctor or nurse). ○ Direct contact, with another reference unit. ○ Indirect contact through Primary Care. ○ Indirect contact through emergency department. ○ Others (open field).	152 (65.8%) 24 (10.4%) 44 (19.0%) 7 (3.0%) 4 (1.7%)
15. Do you know the criteria for Advanced HF?	YES NO	285 (92.5%) 23 (7.5%)
16. Are the criteria for the identification and referral of patients with advanced HF to a referral unit applied?	They DO apply, it is protocolized. They ARE applied by means of interconsultation with the reference unit. NO, I refer the patient to the reference unit to be applied. Others (open field).	105 (34.1%) 151 (49.0%) 41 (13.3%) 11 (3.6%)
17. In your consultation/unit/service, do you have a palliative care unit?	YES NO	147 (47.7%) 161 (52.3%)
17.1. How is palliative care organized in your area?	○ Primary care dependent support. ○ Hospital care dependent support. ○ Support dependent on other home care services. ○ No support.	31 (21.1%) 86 (58.5%) 30 (20.4%) 0 (0%)
18. Is there direct contact with the palliative care unit?	No, it is a referral like any specialist. Yes, there is a direct referral pathway. Yes, there is an indirect route of referral from another agent (e.g., Primary Care, liaison nursing).	142 (46.1%) 131 (42.5%) 35 (11.4%)
19. Do you have a tool to share the patient’s clinical information with Primary Care and/or nursing at your Health Care Center?	Shared single medical record. Access to information through viewers. Others (open field).	194 (63.0%) 91 (29.5%) 23 (7.5%)
20. Is the relationship between the specialized HF consultation and the Health Care Center punctual or structured?	Punctual. Structured.	222 (72.1%) 86 (27.9%)

ARNI: angiotensin receptor–neprilysin inhibitor; BB: beta blockers; CKD: chronic kidney disease; HF: heart failure; HFrEF: heart failure with reduced ejection fraction; LVEF: left ventricular ejection fraction; MRA: mineralocorticoid receptor antagonists; RAASi: renin angiotensin aldosterone system inhibitors; SGLT2i: Sodium-Glucose Cotransporter-2 inhibitors.

**Table 2:** Questionnaire for moderators (post-session).

Areas for Improvement		
Care coordination	Existence of HF referents in Primary Care. Information sharing methods across levels. Cardiology-Primary Care relationship. Cardiology access. Communication channels. Referral and communication protocols.	54 (16.6%) 34 (10.5%) 73 (22.5%) 42 (12.9%) 54 (16.6%) 68 (20.9%)
Diagnosis	Proactive search for at-risk patients. Etiological diagnosis of dyspnea. Natriuretic peptides application. Echocardiogram.	35 (28.5%) 23 (18.7%) 35 (28.5%) 30 (24.4%)
Therapeutic management	Falsely stable patient- IC-BERG Study. Therapeutic plan: optimization. Use of guideline-recommended target doses. Communication that allows individualization of the strategy of initiation and therapeutic sequencing. Coordinated physical training-cardiac rehabilitation plan. Involvement of expert/trained nurses in educational programs for HF patients and caregivers.	39 (17.0%) 58 (25.2%) 35 (15.2%) 40 (17.4%) 18 (7.8%) 40 (17.4%)
Comorbidities	Assessment of frailty. Comprehensive approach and follow-up of fragile patients between Cardiology and primary care. Iron deficiency assessment. Renal function and ion determinations at the initiation or dose modification of RAASi. Patient with HF and atrial fibrillation. Individualized follow-up plan (nursing care, content and frequency of visits according to comorbidities).	27 (21.4%) 18 (14.3%) 25 (19.8%) 13 (10.3%) 10 (7.9%) 33 (26.2%)
Progression of disease	Education of patients and relatives for the early identification of signs/symptoms of decompensation. Fast communication routes for decompensation. Criteria for referral from primary care for reevaluation of the patient for other situations. Direct referral routes from primary care to hospital HF units. Application of the Spanish Society of Cardiology protocol for the transition to discharge (“decalogue”) of the patient admitted for decompensated HF. Application of criteria for the identification and referral of patients with advanced HF to a referral unit. Application of objectives and strategies for the management of patients with terminal HF. Palliative care: support dependent on Primary Care/Cardiology or other home care services	22 (14.6%) 30 (19.9%) 11 (7.3%) 11 (7.3%) 12 (7.9%) 21 (13.9%) 19 (12.6%) 25 (16.6%)

HF: heart failure.

## Data Availability

All data have been included in the manuscript or Supplementary Material.

## Supplementary Materials

The following supporting information can be downloaded at: https://www.mdpi.com/article/10.3390/jcm15072530/s1, Table S1: Pre-session survey (to be answered by participants/attendees); Table S2: Questionnaire for moderators (post-session); Table S3: Post-session questionnaire to be completed by participants; Table S4: Geographical distribution of sessions.
