# Supplementary material for: Improving the Management of Outpatients with Heart Failure the IC-MMERSIVE Project
Source: J Clin Med. 2026 Mar 26;15(7):2530. doi: 10.3390/jcm15072530 (PMC13073573; doi:10.3390/jcm15072530)

Supplementary table S1. Pre-session survey (to be answered by participants/attendees).

Question	Response
**1. When a patient at risk of developing HF is attended, is a proactive search for HF or pre-HF (ACC-AHA Stages A and B) performed?**	• NO<br>• YES<br>If yes, specify how this proactive search is performed (open field question).
**2. In relation to the evaluation of LVEF, do you feel qualified and equipped to perform an echocardioscopy and thus focus the treatment?**	• YES<br>• NO
2.1. If you DO NOT take it, indicate the waiting time to receive this evaluation	o < 15 days.<br>o < 1 month.<br>o 1-3 months.<br>o more than 3 months.
**3. Do you know the 5 questions of the IC-BERG study for the detection/identification of the falsely stable patient?**	• I do NOT know them.<br>• I do know them and apply them in the consultation.<br>• YES, I know them, but I don't apply them/I can't apply them in consultation.<br>- Indicate what barriers you find to apply them (open field).
3.1. If you know them, which of them helps you the most in your real clinical practice (multiple choice)	• Have you stopped doing any activity such as going outside, carrying groceries, climbing the same flights of stairs without stopping, or making things at home, in the last year?<br>• Do you have symptoms of congestion? (Weight gain, jugular ingurgitation, ankle edema, sleeping with more pillows)<br>• Do you need to take ≥ 1 tablet of furosemide a day?<br>• Have you been admitted to or visited the emergency room for HF decompensation in the last year?<br>• Has the NT-proBNP levels changed?
**4. In your consultation, service or unit, do you have a specific protocol with a therapeutic plan to optimize the patient with HF-rEF with the 4 main drugs (ARNI + BB + MRA + SGLT2i)?**	• YES, and it is systematically applied.<br>• YES but I DO NOT apply it systematically.<br>• We do NOT have a specific protocol.
**5. If a protocol is available, does the protocol take into account the use of guideline-recommended target doses?**	• YES<br>• NO<br>• Not applicable: we do not have a protocol.
**6. Do you use guideline-recommended target doses in clinical practice? If they are not used, indicate the reasons** (multiple choice)	• Yes, I use guideline-recommended target doses.<br>• I do not prescribe them because I do not think they are essential.<br>• I do not prescribe them because our protocol does not contemplate them.<br>• I do not use them because the patient cannot tolerate them.<br>• I do not use them: they are referred to another unit, and I do not perform the drug titration.
**7. In your consultation, is nursing trained in educational programs for patients and caregivers involved in HF?**	• NO, nursing is not involved in educational programs in my consultation.<br>• Nursing is involved and nursing care (assessment, health education and advice) is applied to all or most HF patients.<br>• Nursing is involved but nursing care (assessment, health education and advice) is NOT applied to all or most HF patients.
7.1. What barriers do you think exist for its application? (multiple choice)	• Education.<br>• Consultation time.<br>• The assigned nursing tasks do not have this.<br>• The patient is referred to a different unit/service where it is performed.<br>• Others (open field).
**8. Is frailty assessed in patients over 75 years of age in your consultation?**	• YES, it is assessed WITH previously validated scales.<br>• YES, it is assessed but WITHOUT scales: it is a subjective assessment by the cardiologist and/or nurse.<br>• NOT usually evaluated.
**9. If the patient is considered to be fragile, how is follow-up performed?**	• The patient is referred to a specialized unit (Internal Medicine or Geriatrics).<br>• Follow-up continues in Cardiology.<br>• Follow-up is referred to Primary Care.
**10. Returning to the protocol or the assessment of the patient with HF. Is iron deficiency taken into account? Is it done in a structured way?**	• NO<br>• YES
10.1. If yes, how often?	o It is not structured and depends on the patient and the appointment capacity of the consultation.<br>o 6 months.<br>o 1 year.<br>o More than 1 year.<br>o Only if symptoms.
**11. What do you do with patients once iron deficiency has been diagnosed and treated?**	• Routine follow-up of patients with HF.<br>• Routine follow-up and fecal occult blood screening.<br>• Referral or consultation for the study of iron deficiency in Internal medicine or Digestive specialist.<br>• Individualization according to the patient and his/her characteristics.

**12. Does the treatment protocol take into account the need to determine renal function and ions when initiating and/or modifying doses of RAASi? Are patients with CKD considered in a special way?**	• Yes, it is included in the protocol and considers patients with CKD in a special way.<br>• Yes, it is included in the protocol, although patients with CKD are not considered in a special way.<br>• I do not have a protocol, but I do and I take into account patients with CKD in a special way.<br>• I do not have a protocol and I am not specifically prepared to do it.<br>- Indicate what barriers you encounter (Open field)
**13. Is there a follow-up protocol in your consultation/unit/service that includes nursing care and the frequency and content of follow-up visits according to comorbidities and/or frailty and the relationship with (apply as the case may be) Cardiology, Primary Care, Internal Medicine, Geriatrics or other specialties?**	• YES<br>• NO
**14. Does the patient treated in your consultation have fast communication channels in case of decompensation?**	• YES<br>• NO
**14.1.** If fast routes are available, who does the patient contact? Is contact direct or indirect through another agent (e.g. Primary Care or Emergency department)?	o Direct contact with my consultation (doctor or nurse).<br>o Direct contact, with another reference unit.<br>o Indirect contact through Primary Care.<br>o Indirect contact through emergency department.<br>o Others (open field).
**15. Do you know the criteria for Advanced HF?**	• YES<br>• NO
**16. Are the criteria for the identification and referral of patients with advanced HF to a referral unit applied?**	• They DO apply, it is protocolized.<br>• They ARE applied, by means of interconsultation with the reference unit.<br>• NO, I refer the patient to the reference unit to be applied.<br>• Others (open field).
**17. In your consultation/unit/service, do you have a palliative care unit?**	• YES<br>• NO
17.1. How is palliative care organized in your area?	o Primary care dependent support.<br>o Hospital care dependent support.<br>o Support dependent on other home care services.<br>o No support.
**18. Is there direct contact with the palliative care unit?**	• No, it is a referral like any specialist.<br>• Yes, there is a direct referral pathway.<br>• Yes, there is an indirect route of referral from another agent (e.g. Primary Care, liaison nursing).
**19. Do you have a tool to share the patient's clinical information with Primary Care and/or nursing at your Health Care Center?**	• Shared single medical record.<br>• Access to information through viewers.<br>• Others (open field).
**20. Is the relationship between the specialized HF consultation and the Health Care Center punctual or structured?**	• Punctual.<br>• Structured.

ARNI: angiotensin receptor–neprilysin inhibitor; BB: beta blockers; CKD: chronic kidney disease; HF: heart failure; HF-rEF: heart failure with Reduced ejection fraction; LVEF: left ventricular ejection fraction; MRA: mineralocorticoid receptor antagonists; RAASi: renin angiotensin aldosterone system inhibitors; SGLT2i: Sodium-Glucose Cotransporter-2 inhibitors.

Supplementary table S2. Questionnaire for moderators (post-session)

The moderator, in consensus with the participants, could identify one or more areas for improvement and one or more lines within the selected area of action.<br>*It was mandatory to indicate at least* ***one area of action*** *and* ***one line within that area of action****.*	
**Areas for improvement**	
**Care coordination**	• Existence of HF referents in Primary Care<br>• Information sharing methods across levels<br>• Cardiology-Primary Care relationship<br>• Cardiology access<br>• Communication channels<br>• Referral and communication protocols
**Diagnosis**	• Proactive search for at-risk patients.<br>• Etiological diagnosis of dyspnea.<br>• Natriuretic peptides application.<br>• Echocardiogram.
**Therapeutic management**	• Falsely stable patient- IC-BERG Study.<br>• Therapeutic plan: optimization.<br>• Use of guideline-recommended target doses.<br>• Communication that allows individualization of the strategy of initiation and therapeutic sequencing.<br>• Coordinated physical training-cardiac rehabilitation plan.<br>• Involvement of expert/trained nurses in educational programs for HF patients and caregivers.
**Comorbidities**	• Assessment of frailty.<br>• Comprehensive approach and follow-up of fragile patients between Cardiology and primary care.<br>• Iron deficiency assessment.<br>• Renal function and ion determinations at the initiation or dose modification of RAASi.<br>• Patient with HF and atrial fibrillation.<br>• Individualized follow-up plan (nursing care, content and frequency of visits according to comorbidities).
**Progression of disease**	• Education of patients and relatives for the early identification of signs/symptoms of decompensation.<br>• Fast communication routes for decompensation.<br>• Criteria for referral from primary care for reevaluation of the patient for other situations.<br>• Direct referral routes from primary care to hospital HF units.<br>• Application of the Spanish Society of Cardiology protocol for the transition to discharge ("decalogue") of the patient admitted for decompensated HF.<br>• Application of criteria for the identification and referral of patients with advanced HF to a referral unit.<br>• Application of objectives and strategies for the management of patients with terminal HF.<br>• Palliative care: support dependent on Primary Care/Cardiology or other home care services

HF: heart failure.

Supplementary table S3. Post session questionnaire to be completed by participants.

**Section 1.**	
**In case your consultation, service or unit is in one of these scenarios, answer the questions included in this section.**<br>**If it does not apply to the situation of your consultation, service, or unit, you can move on to the next section.**	• Scenario 1- There is NO specific protocol with a therapeutic plan to optimize the patient with HF-rEF with the 4 main drugs (ARNI + BB + MRA + SGLT2i).<br>• Scenario 2 - The protocol available does NOT consider the use of guideline-recommended target doses.<br>• Scenario 3- After the session, clear points of improvement have been identified in relation to the optimization of treatment.
**Do you think it is necessary to have a protocol for initiating and/or optimizing treatment with the 4 main drugs (ARNI + BB + MRA + SGLT2i) and try to titrate up to guideline-recommended target doses?**	• YES<br>• NO
What elements would be essential to be able to carry out this plan? What points of improvement would you add?	○ Respond with 4-5 key ideas (open field).
**Section 2**	
**In case that frailty is NOT assessed in patients over 75 years of age in your consultation or that after the session clear points of improvement have been identified, answer the question in this section.**<br>**If it does not apply to the situation of your consultation, service, or unit, you can move on to the next section.**	
**Do you think it is necessary to make an individual plan for the frail patient with HF?**	• YES<br>• NO
On what basis would you support this plan? How would you make a realistic approach? What points of improvement would you add?	○ Respond with 4-5 key ideas (open field).
**Section 3**	
**If your consultation/unit/service is in one of these scenarios, answer the questions in this section.**<br>**If it does not apply to the situation of your consultation, service, or unit, you can move on to the next section.**	• There is NO follow-up protocol that includes nursing care and the frequency and content of follow-up visits based on comorbidities and/or frailty and the relationship with (apply as the case may be) Cardiology, Primary Care, Internal Medicine, Geriatrics or other specialties.<br>• The patient attended in the consultation does not have fast communication channels for decompensation.<br>• After the session, clear points for improvement are identified, in relation to the situations described above.
**Do you think an individualized follow-up plan is necessary depending on the patient's phenotype?**	• YES<br>• NO
On what basis would you support this plan? How would you make a realistic approach? What points of improvement would you add?	○ Respond with 4-5 key ideas (open field).
**Section 4**	
**How do you think that, in your setting, a fast communication route could be implemented, or improve current communication? (multiple response)**	• No face-to-face interconsultations.<br>• Calls to a personalized HF number.<br>• Calls to a general cardiologist.<br>• Other, comment (open field).

ARNI: angiotensin receptor–neprilysin inhibitor; BB: beta blockers; HF: heart failure; MRA: mineralocorticoid receptor antagonists; SGLT2i: Sodium-Glucose Cotransporter-2 inhibitors.

Supplementary table S4. Geographical distribution of sessions.

Province	N (%)	Province	N (%)
Alicante	1 (0.7)	Lleida	1 (0.7)
Almeria	4 (2.8)	Lugo	2 (1.4)
Asturias	5 (3.5)	Madrid	19 (13.2)
Badajoz	2 (1.4)	Málaga	6 (4.2)
Barcelona	28 (19.4)	Murcia	1 (0.7)
Bizkaia	6 (4.2)	Navarra	3 (2.1)
Burgos	2 (1.4)	Ourense	3 (2.1)
Cadiz	5 (3.5)	Palmas, Las	6 (4.2)
Castellon	1 (0.7)	Pontevedra	6 (4.2)
Ciudad Real	1 (0.7)	Rioja, La	1 (0.7)
Cordoba	2 (1.4)	Salamanca	1 (0.7)
Coruña, A	4 (2.8)	Santa Cruz de Tenerife	4 (2.8)
Gipuzkoa	2 (1.4)	Seville	7 (4.9)
Granada	2 (1.4)	Tarragona	4 (2.8)
Huelva	2 (1.4)	Toledo	1 (0.7)
Jaen	1 (0.7)	Valencia	4 (2.8)
Leon	3 (2.1)	Zaragoza	4 (2.8)

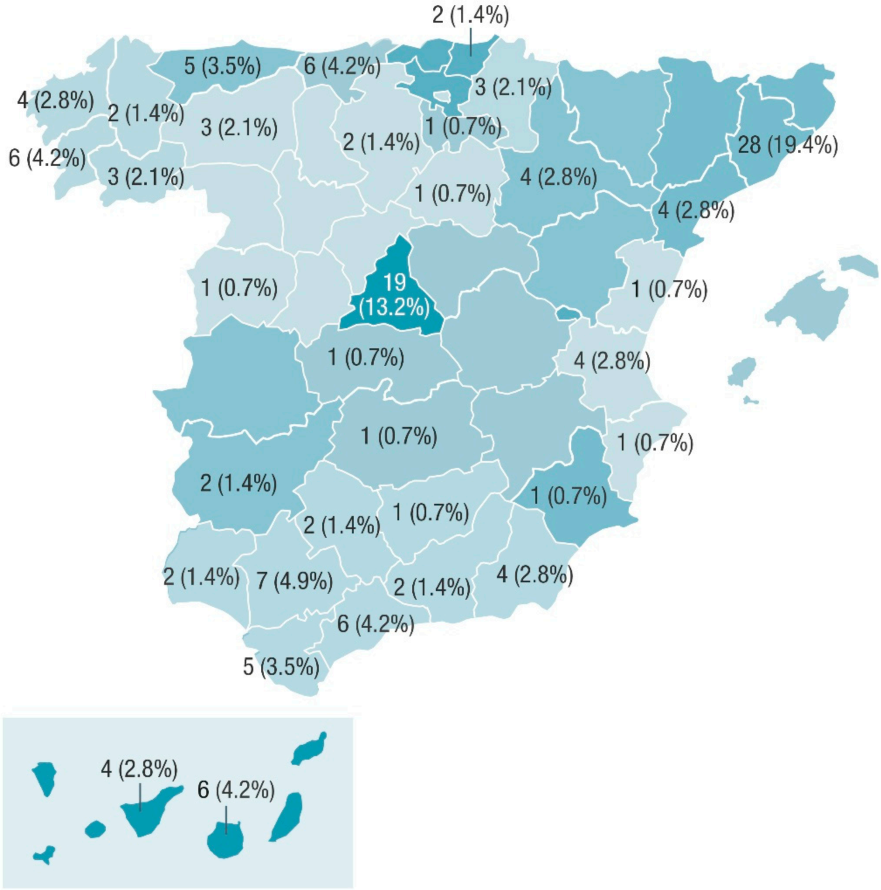

Supplement: Supplementary file 1 [file jcm-15-02530-s001.zip › jcm-4208242-supplementary.pdf]
